# Synthesis and Characterization of Graphene Oxide and Reduced Graphene Oxide Composites with Inorganic Nanoparticles for Biomedical Applications

**DOI:** 10.3390/nano10091846

**Published:** 2020-09-15

**Authors:** Joanna Jagiełło, Adrian Chlanda, Magdalena Baran, Marcin Gwiazda, Ludwika Lipińska

**Affiliations:** 1 Department of Chemical Synthesis and Flake Graphene, Łukasiewicz Research Network-Institute of Electronic Materials Technology, 133 Wólczyńska Str., 01-919 Warsaw, Poland; adrian.chlanda@itme.edu.pl (A.C.); magdalena.baran@itme.edu.pl (M.B.); st.m.gwiazda@gmail.com (M.G.); ludwika.lipinska@itme.edu.pl (L.L.); 2 Faculty of Material Science and Engineering, Warsaw University of Technology, 141 Wołoska Str., 02-507 Warsaw, Poland

**Keywords:** reduced graphene oxide, inorganic nanoparticles, composites, biomaterials

## Abstract

Graphene oxide (GO) and reduced graphene oxide (RGO), due to their large active surface areas, can serve as a platform for biological molecule adhesion (both organic and inorganic). In this work we described methods of preparing composites consisting of GO and RGO and inorganic nanoparticles of specified biological properties: nanoAg, nanoAu, nanoTiO_2_ and nanoAg_2_O. The idea of this work was to introduce effective methods of production of these composites that could be used for future biomedical applications such as antibiotics, tissue regeneration, anticancer therapy, or bioimaging. In order to characterize the pristine graphene materials and resulting composites, we used spectroscopic techniques: XPS and Raman, microscopic techniques: SEM with and AFM, followed by X-Ray diffraction. We obtained volumetric composites of flake graphene and Ag, Au, Ag_2_O, and TiO_2_ nanoparticles; moreover, Ag nanoparticles were obtained using three different approaches.

## 1. Introduction

In the last decade, the properties of graphene materials have been intensively studied in the scientific community in order to develop their potential in various biomedical applications. Graphene is a single layer (in thickness) of carbon atoms arranged in hexagonal rings of an aromatic electron structure. Graphene oxide and reduced graphene oxide are the graphene derivatives characterized by the presence of oxygen functional groups on the graphene surface (more in graphene oxide (GO) than in reduced graphene oxide (RGO)) and, therefore, by the presence of defects in the graphene hexagonal rings as well. Such disorder in the graphene structure leads to many unique properties of GO and RGO.

Due to the presence of oxygen groups bonded to the surface, GO is an electrical insulator (ideal graphene is an excellent semiconductor). GO is highly hydrophilic and therefore has great wettability. The surface of both GO and RGO is susceptible to modifications with different molecules, including biological ones, which allows composites with strictly controlled and desired properties to be synthesized. GO and RGO, due to their large active surface areas, can serve as a platform for biological molecules (both organic and inorganic) to be safely introduced into the organism without the risk of uncontrolled and undesirable spreading into the surrounding tissues. This is a great advantage, for example, in the case of anticancer therapy, where cytotoxic compounds are introduced into the tumor [1,2,3,4,5].

In many biomedical applications, it is crucial to use materials that are nontoxic to human cells, in terms of this requirement GO and slightly reduced GO seem to be suitable materials. The biocompatibility of these two graphene derivatives has been confirmed by many publications in the literature [6]. This aspect of GO and RGO materials presented in this study was also previously investigated by us with human umbilical cord mesenchymal stem cells (hUC-MSCs) [7]. The overriding conclusion from this study was that none of the tested materials (GO and RGO) were characterized with a negative impact in the cellular viability, proliferation, morphology, or gene expression. It was also found that the cytotoxicity depends on the size of the graphene flakes and the content of the oxygen functional groups: smaller flakes (about 0.2–2 μm of lateral size) and highly reduced graphene oxide (approximately 10% of remaining oxygen atoms) do have a toxic effect. Therefore, in this work we used RGO with a higher oxygen content and GO with larger flakes that were nontoxic to hUC-MSCs.

There are two main synthesis methods of graphene oxide: Hummers and Offeman [8] and Marcano and Tour [9]. The first one consisted of adding potassium manganate (KMnO_4_) as an oxidant to the reaction mixture in the presence of concentrated sulfuric acid (VI) and NaNO_3_ salt. Marcano and Tour proposed an improved method of graphite oxidation synthesis involving the elimination of sodium nitrate (V) from the reaction mixture, increasing the amount of potassium manganate (VII) and carrying out the reaction in the presence of concentrated acids: H_2_SO_4_ and H_3_PO_4_ in a volume ratio of 9:1. As a result, GO with a higher content of oxygen functional groups can be obtained. On the basis of these methods, numerous modifications are made to the synthesis of graphene oxide: partial oxidation to prepare GO of C/O atomic ratio between 12 and 3 [10], double oxidation leading to a high carboxyl groups concentration [11]; the use of different concentration of nitric acid (from 50% to 98%) leading to GO structure of C/O atomic ratio between 6.5 and 2.8 [12] and fast, 1-h oxidation [13].

In this work, we used Ag, Ag_2_O, Au, and TiO_2_ nanoparticles to create composites with GO and RGO. Silver nanoparticles (AgNPs) were chosen because of their antibacterial properties—AgNPs have been shown to be effective against gram-positive and gram-negative bacteria, as well as fungi and viruses. Currently, nanosilver is used in many fields of industry, most widely in the production of dressings for accelerated wound healing and medical implants, as well as in cosmetics, dentistry, water filtration, or textile production. The mechanism of its action is based on its ability to interact with the thiol groups of bacterial cell walls and to disrupt the cell membrane. This leads to the denaturation and inactivation of enzymatic proteins that are a part of the respiratory chain, the formation of reactive oxygen species (ROS) and, consequently, the appearance of oxidative stress of the cell [14].

Gold nanoparticles (AuNPs) are widely used in such areas of medicine as diagnostics, cancer treatment and drug delivery systems. The ability of nanogold to enhance light scattering and absorption due to surface plasmon resonance is used in cancer diagnosis and therapy. Binding of nanogold to ligands allows the specific targeting of biomarkers for imaging and cancer detection. In addition, it can convert absorbed light into localized heat energy, which can be used in laser photothermal therapy. In addition, it has been shown that AuNPs can have a positive effect on the differentiation (osteogenesis) of hMSCs. The influence of AuNPs’ shape and size on the survival, proliferation, and expression of osteogenic gene markers has been demonstrated [15,16,17].

Nano-titanium oxide, in addition to its antibacterial properties which have been used in decontamination preparations, is widely used in tissue engineering as part of biocomposites and surface coatings. The topography of titanium’s surface plays a very important role in biomedical applications. As the rough and porous morphology of Ti imitates native bone architecture, it enhances osteoblasts adhesion, maturation and bone formation. The distribution of charge and the surface chemistry of titanium materials are also important and can be one of the key factors inducing stem cells differentiation to osteoblasts. Much work has focused on the use of nano-TiO_2_ as a photosensitizer in the treatment of cancer due to its high photocatalytic activity, low toxicity, and high photostability [18,19].

Literature reports indicate the possibility of using nano-Ag_2_O in the treatment of venous ulceration. Silver oxide used as an ingredient in dressing ointment resulted in improved microcirculation and wound healing [20]. Considering the possible anti-inflammatory properties and accelerated wound healing, we also decided to prepare a nano-Ag_2_O composite with flake graphene as a potential material in tissue engineering.

The other approaches related to the fabrication of the antibacterial composites based on the graphene oxide contained the addition of graphitic carbon nitride (GO/g-C_3_N_4_) [21], zinc oxide (ZnO) [22] and Ag/Cu bimetallic nanoparticles (NPs) [23]. Other kinds of GO composites are those with the anti-inflammatory properties. They were formed using Fe_2_(MoO_4_)_3_ nanorods [24], aerogel (GA)-supported metal-organic framework (MOF) particles [25] and polyoxotungstate [26]. There is also another great research area that uses graphene composites: regenerative tissue engineering. The examples can be calcium silicate—graphene composites [27] and graphene oxide-calcium phosphate nanocomposites [28] for osteogenic and angiogenic differentiation of human mesenchymal stem cells. For neural tissue engineering, RGO is commonly used due to its electrical properties and one of the described materials is RGO/TiO_2_ for photo stimulation of neural stem cells [29].

Knowing that both morphology and chemical composition of composites for potential biomedical applications may result in different properties of the obtained materials, we decided to perform a detailed and systematic study addressing this issue. For this purpose, this manuscript is focused exclusively on the material aspect: the synthesis and characterization of graphene-nanoparticles composites. This work, for the first time, provides a comprehensive study concerning both GO and RGO composites with various types of inorganic nanoparticles of a specified biological activity. We plan to perform and discuss the biological assessment of the aforementioned composite materials in the future.

## 2. Materials and Methods

For the experiments, the following chemical compounds were used: graphite (Asbury Carbons, Asbury, NJ, U.S.; with a particle diameter of 300–425 µm), sulfuric acid (Poch S.A., Gliwice, Poland, 96–98% pure p. a.), orthophosphoric acid (Chempur, Piekary Śląskie, Poland, pure p. a.), potassium permanganate (Chempur, Poland, pure p. a.), L(+)-ascorbic acid (Poch S.A, pure p. a.), sodium hypophosphite monohydrate (Chempur, pure p. a.), hydrochloric acid (Chempur, pure p. a.), perhydrol (Chempur, pure p. a.), and ethanol (Poch S.A., 96%, pure p. a.). The compounds used for nanoparticle synthesis included NaOH (Chempur, pure p. a.), NaBH_4_ (Sigma Aldrich, Schnelldorf, Germany, 99%), Polyphenon PP60 (Sigma Aldrich), AgNO_3_ (pure p. a.), HAuCl_4_·3H_2_O (Roth, ≥ 99.5%), titanium isopropoxide (Sigma Aldrich, 97%), sodium citrate (Poch S.A., pure), and absolute ethanol (Merck Millipore, Darmstadt, Germany).

The morphology of the materials was examined by scanning electron microscopy SEM (Auriga CrossBeam Workstation, Carl Zeiss) and atomic force microscopy AFM (Dimension Icon, Bruker; with tapping mode and OTESPA R3 scanning probe, Bruker). The chemical structure was studied with Raman Spectroscopy (Renishaw Invia, excitation laser source: 532 nm; laser power: < 1 mW), XPS Spectroscopy (UHV Multichamber XPS System, Prevac; with Al Kα X-ray source (1486.6 eV)) and X-ray diffraction XRD (Rigaku Diffractometer, Japan; with Cu Kα anode of 8.038 keV, UC=C40 kV, IC=C30 mA, scanning speed: 2 deg/min, sampling density: 0.02 deg). For XRD, Raman and XPS measurements, the samples were prepared in the form of powders. AFM, SEM and EDS measurements were conducted on layers of the samples placed on a silicon substrate, without sputtering.

### 2.1. GO Preparation

The GO was prepared by the modified Marcano method [9]. In brief: 3 g of graphite flakes (with the average size of 300–425 µm) were added gradually to a reactor containing 360 mL of concentrated sulphuric acid and 40 mL of orthophosphoric acid. After that, 18 g of potassium permanganate were slowly added to the graphite. The oxidation process was conducted for a few hours and it was stopped by the addition of deionized water and finally—3 mL of perhydrol (30% H_2_O_2_; Chempur, pure p.a.). The water suspension of such obtained graphite oxide was left to sediment. The purifying process was carried out with a custom made microfiltration system. Due to specific shearing forces acting on the GO flakes during the purification process, the flakes were exfoliated at the same time.

The chemical formula of GO was assumed as “C_2_O”, where there is one oxygen atom per two carbon atoms, which gives a molar mass of 40 g/mol. Such structure is in agreement with the information obtained from the XPS measurement and with the literature as well [30].

### 2.2. RGO Preparation

The RGO was prepared via a “green” reduction process by using L(+)-ascorbic acid (C_6_H_8_O_6_) as a reducing agent. An L-ascorbic acid solution was added to the previously prepared GO water suspension with GO: L-ascorbic acid molar ratio of 1:4. The mixture was reduced for 3.5 h at a temperature of 95 °C with constant stirring. The GO molar mass was assumed to be 40 g/mol with respect to the C_2_O chemical formula. The prepared RGO was then filtered with ultrapure water to remove the remaining ions. Gao et al. (2010) postulated a reduction mechanism proceeded via a two-step S_N_2 nucleophilic reaction followed by another one-step thermal elimination [31].

### 2.3. Preparation of Graphene Composites with Ag, Au, Ag_2_O, and TiO_2_ Nanoparticles

#### 2.3.1. Composites with Nano-Ag

Three different reducing agents were used to reduce Ag^+^ ions to Ag nanoparticles: L(+) ascorbic acid (Ag[KA]), sodium borohydride (Ag[BS]) and polyphenols from green tea (Ag[PP60]).

##### Composites with Nano-Ag[KA]

**Figure 1:**
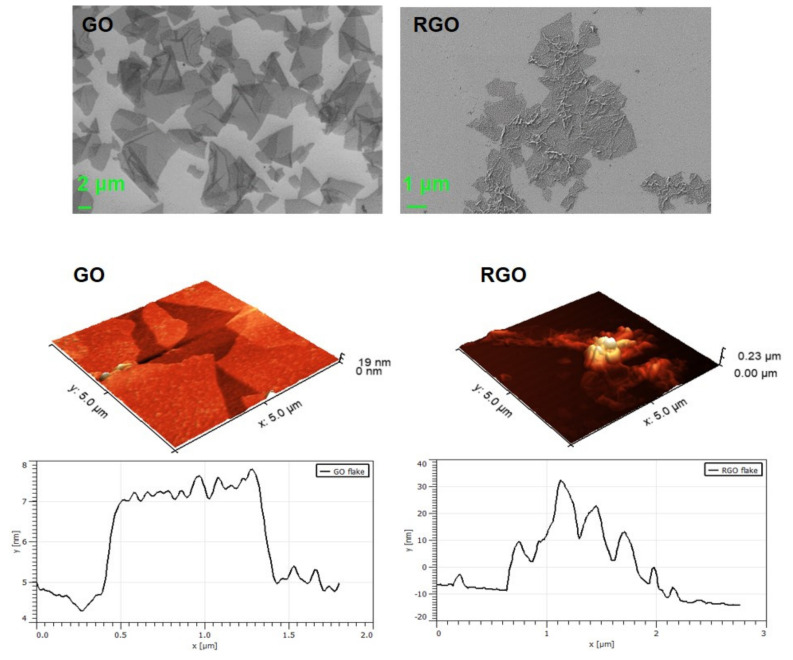
SEM images of graphene oxide (GO) and reduced graphene oxide (RGO) (deposited on Si substrate) and AFM thickness profile of GO and RGO.

To prepare the composite of GO with nano-Ag obtained with L-ascorbic acid (GO Ag[KA]), a silver nitrate (V) solution was added to the aqueous suspension of GO while stirring and after a few minutes an aqueous solution of L-ascorbic acid was added dropwise. The reduction mechanism is shown in Figure 1. The molar ratio of GO to silver nitrate (V) was 1:0.08, while the molar ratio of silver nitrate (V) to L-ascorbic acid was equal to 1:2. Adding the compounds in this order resulted in a homogeneous coating of graphene oxide with silver particles. The reaction was carried out at room temperature to ensure that the reduction would involve silver ions only, without disturbing the graphene oxide structure. The mixture was left for 24 h with vigorous stirring and was then dialyzed for 72 h to remove the residual ions.

The RGO composite with Ag[KA] (RGO-Ag[KA]) was prepared using the above recipe for GO-Ag[KA], followed by the addition of L-ascorbic acid to finally reduce the GO. The reaction was performed for 3.5 h at 95 °C. The GO: L-ascorbic acid molar ratio was 1:4. After the reaction was completed, the material was purified by dialysis. The two-step process was carried out to obtain a good distribution of silver nanoparticles on RGO flakes. Because GO flakes are well dispersed, firstly, nano-Ag was precipitated on these non-agglomerated flakes. After that, the GO reduction causing also partial agglomeration of the flakes was conducted (with the use of ascorbic acid and the higher temperature) without negative influence on the distribution of the nanoparticles on RGO flakes.

##### Composites with Nano-Ag[BS]

A reaction between silver nitrate and sodium borohydride in a water solution was described by Sobczak-Kupiec et al. (2011) [32]. In brief, the reaction can be written as follows:

AgNO_3_ + NaBH_4_ + 3H_2_O → Ag + NaNO_3_ + H_3_BO_3_ + 3.5H_2_

To obtain the GO-Ag [BS] composite, silver nitrate (V) was added successively to the aqueous GO suspension, followed by sodium borohydride. The molar ratio of GO (MC=C40 g/mol) to silver was 1:0.08 and the molar ratio of silver nitrate (V) to sodium borohydride was 1:2. NaBH_4_ creates a reductive and alkaline environment, causing deprotonation of carboxylic groups, which renders more negative zeta potential and therefore improves the GO flakes stability [33]. However, salt type and ionic strength have a significant effect on GO stability. Here, the presence of Na^+^ ions could compensate for the effect of the decrease in zeta potential, finally leading to the flakes agglomeration. The hydrodynamic dimension depends on the type of ions present in the GO sample and is bigger for multivalent ions than monovalent ones; it also depends on the concentration of these ions [34].

The reaction was continued for 24 h at a room temperature with continuous stirring. The material was then dialyzed for 72 h to remove the remaining ions.

The RGO-Ag[BS] composite was obtained through the preparation of the GO-Ag[BS] composite followed by the addition of L-ascorbic acid to reduce the GO. The reaction was carried out for 3.5 h at 95 °C. The GO: L-ascorbic acid molar ratio was 1:4. After the reaction was completed, the material was dialyzed for 72 h.

##### Composites with Nano-Ag[PP60]

To obtain the GO-Ag[PP60] composite, an aqueous AgNO_3_ solution was added to the aqueous suspension of GO at a molar ratio of 1:0.08. Then, the polyphenol PP60 solution was added dropwise in a 1:1 molar ratio with AgNO_3_. The reagents were vigorously stirred on a magnetic stirrer for 24 h at room temperature. The material was purified by dialysis.

The procedure for obtaining the RGO composite with Ag[PP60] nanoparticles consisted of producing the GO-Ag[PP60] composite (as described above) and adding L-ascorbic acid to reduce the GO. The reaction was carried out for 3.5 h at 95 °C. The GO:ascorbic acid molar ratio was 1:4. After the reaction was completed, the material was dialyzed.

#### 2.3.2. Composites with Nano-Au

To prepare the GO-Au composite, an aqueous solution of HAuCl_4_·3H_2_O was added dropwise to the GO water suspension at a GO:HAuCl_4_ molar ratio of 1:0.08. The mixture was stirred on a magnetic stirrer for 30 min at room temperature (to obtain a homogeneous mixture of GO flakes and Au^3+^ ions), after which the temperature was increased to 80 °C to provide the conditions needed for Au^3+^ reduction. Then, an aqueous solution of sodium citrate (3-hydrated) was added dropwise at a molar ratio with HAuCl_4_ of 1:0.17. The reaction continued for 1 h at 80 °C while being stirred. A composite with a purple glow was formed, proving the formation of gold nanoparticles. The material was dialyzed to remove the remaining ions. The mild reaction conditions did not reduce the GO, only Au^3+^ to Au^0^.

To prepare the RGO-Au composite, L-ascorbic acid was added to the previously prepared GO-Au composite. The reduction was carried out on a magnetic stirrer for 3.5 h at 95 °C and purified by dialysis.

#### 2.3.3. Composites with Nano-Ag_2_O

The GO-Ag_2_O composite was obtained by adding silver nitrate (V) solution followed by sodium hydroxide solution to the aqueous GO suspension. This order of adding compounds was used because by adding NaOH first would cause GO agglomerates to form (in the highly alkaline environment) and the Ag nanoparticles to be poorly distributed on the flakes. The addition of AgNO_3_ to GO before NaOH provides less alkaline conditions due to the presence of strong acid salt. The molar ratio of GO to silver nitrate (V) was 1:0.08 and the molar ratio of silver nitrate (V) to sodium hydroxide was 1:1. The reaction was carried out at room temperature for 24 h with continuous stirring. The resulting material formed a stable aqueous suspension that was dialyzed to remove residual ions.

The RGO-Ag_2_O composite was produced by reducing GO with L-ascorbic acid as described in Section 2.2. An aqueous solution of AgNO_3_ and NaOH was then added to RGO to precipitate Ag_2_O nanoparticles. The reactions were carried out at room temperature for 24 h followed by purification with dialysis.

#### 2.3.4. Composites with Nano-TiO_2_

To obtain the GO-TiO_2_ composite, titanium isopropoxide was added dropwise to a small amount of absolute ethanol and then added slowly to the aqueous GO suspension under vigorous stirring. During the addition of GO, a white TiO_2_ precipitate formed. Proper amounts of the compounds were used to obtain a molar GO:TiO_2_ ratio of 1:0.08.

The RGO-TiO_2_ composite was prepared by reducing the GO with L-ascorbic acid and purifying it by dialysis. The appropriate amount of titanium isopropoxide (standard 1:0.08 molar ratio) was then added dropwise to a small amount of absolute ethanol, after which it was added slowly to the RGO suspension under vigorous stirring. The sample was stirred for 24 h in order to precipitate out TiO_2_ particles.

## 3. Results and Discussion

### 3.1. GO and RGO Characterization

#### 3.1.1. SEM and AFM Visualization

The images from the SEM (Figure 1) show the morphology and the sizes of the resulting GO and RGO flakes, which was 10–20 μm and a few micrometers, respectively. The petals form thin “curtains” that wrinkle if they are large enough. The SEM images indicate the formation of small agglomerates of RGO, which is not observed for GO flakes. This is the result of the smaller number of oxygen functional groups on the RGO surface. These oxygen groups are responsible for stabilizing the material: by forming both hydrogen bonds between groups of adjacent flakes (protonated form) and repulsive interactions between charged flakes. In RGO there is a small amount of them so the stabilization does not occur as in GO. The π–π stacking (π–π interaction of aromatic structure) of RGO flakes can be also responsible for their agglomeration. It is difficult to estimate the size of RGO flakes because they agglomerate and overlap.

The working principle of AFM—based on mechanical contact between the microscope’s scanning probe and tested surface allowed for the quantification of average thickness of GO and RGO flakes. AFM was operating in tapping mode, which enabled an acquisition of high resolution topographical images, from which the flake thickness was estimated. The average GO flakes thickness was estimated at 1.2 ± 0.2 nm. Knowing that the thickness of pure graphene (exfoliated, CVD) is between 0.4–1.7 nm [35], and taking into account the presence of functional groups in the GO structure (which are a component of the total thickness), we can conclude that the GO we produced was characterized with almost complete exfoliation into stacks composed of one to three flakes. The measured thickness of RGO material was in a range of 20–180 nm (Figure 1). Reduced graphene oxide was made from GO, the thickness of which was determined by the AFM. It is worth underlining that because of the strong tendency of RGO flakes to create agglomerates it is difficult to estimate the thickness on individual flakes.

#### 3.1.2. XPS Spectroscopy

**Figure 2:**
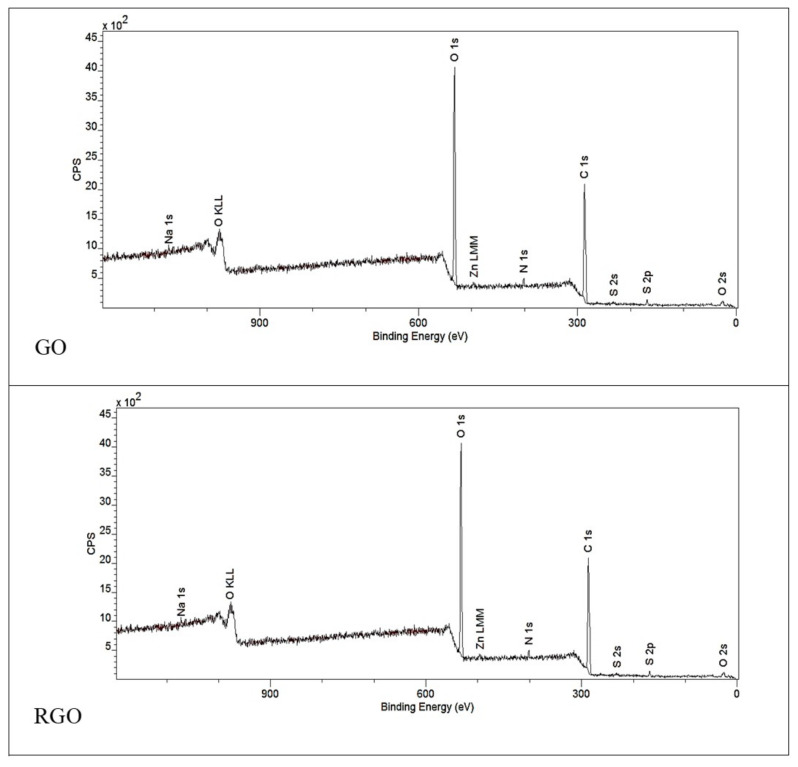
XPS survey spectra of GO and RGO.

The XPS survey spectra (Figure 2) and the information gathered in Table 1 describe the elemental composition of the GO and RGO samples. The most prominent is the difference of oxygen content for these materials, indicating the removal of oxygen groups and the reduction of the initial GO. The oxygen content in GO is 33% with a C:O atomic ratio of 1.98; for RGO it is 17.8% with a C:O atomic ratio of 4.47. The presence of other elements visible in the samples can be attributed to the residue of post-reaction ions.

**Figure 3:**
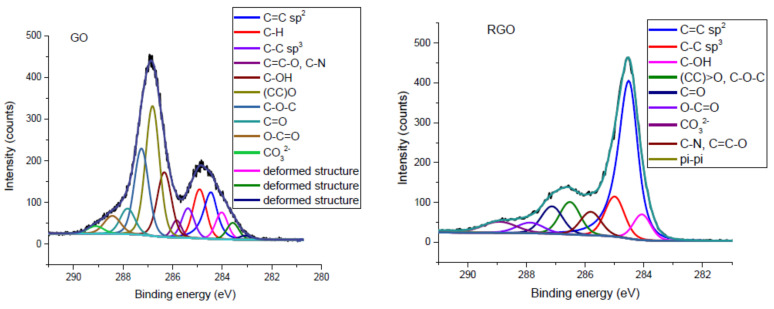
XPS C1s spectra of GO and RGO and their deconvolution.

Figure 3 presents the XPS C1s spectra of the GO and RGO samples. The spectra can be deconvoluted into bands, corresponding to carbon atoms with sp^2^ (C=C) and sp^3^ (C–C) hybridization, hydroxyl/phenyl (C–OH), epoxy ([CC] > O), ether (C–O–C), carbonyl (CC=CO), and carboxyl (O–C=O). Carbonate ions (CO_3_^2−^) and defective carbon structures have also been detected. Table 2 presents detailed information on the functional groups with their percentage content in tested samples. The results indicate that epoxy groups were the most numerous among the functional groups on the GO surface (~25%). Hydroxyl (~13%) and ether (~17%) groups also had a large share. Carboxyl groups represented only 4% of the atoms, which is to be expected, as they are primarily found on the edges of the flakes.

The spectra from the C1s region of GO and RGO display very significant differences, mainly concerning the ratio of C=C (sp^2^) band intensity to those corresponding to oxygen groups—especially hydroxyl (C–OH), epoxy ([CC] > O), and ether (C–O–C). In RGO, as a result of the reduction, the C=C double bond structure was partially restored: from 10.22% for GO to 43.70% (4.3-fold increase). The XPS measurement shows that functional oxygen groups were less frequent in RGO than GO. Moreover, the sum of ether and epoxy groups in GO was 41.82%, while in RGO it was 10.40%. The results of XPS spectroscopy confirmed that GO flakes were successfully reduced using L-ascorbic acid.

#### 3.1.3. X-ray Diffraction (XRD)

**Figure 4:**
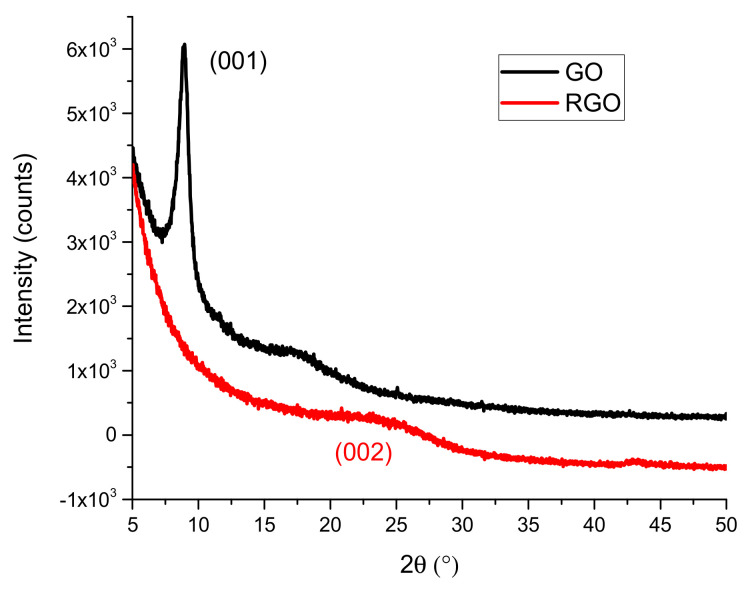
XRD spectra of the GO and RGO samples.

The GO and RGO powders obtained by freeze-drying were characterized with the XRD method. In Figure 4, the characteristic carbon peak for GO (001) at 2θ ≈ 9° is shown. GO has an order of layers, where the interplanar distance (d) is 9.98 Å and the average number of layers is 10. After GO reduction, the reflection (001) disappears and (002) reappears at 2θ ≈ 24° [44,45]. This RGO band is blurred, which indicates that the carbon planes were poorly packaged. The interplanar distance (d) for RGO was 3.6 Å (this is the average value due to the wide band (002)), and the number of layers in the package was 3.4. The higher d value in GO is due to the presence of numerous oxygen functional groups between the graphene layers.

#### 3.1.4. Raman Spectroscopy

**Figure 5:**
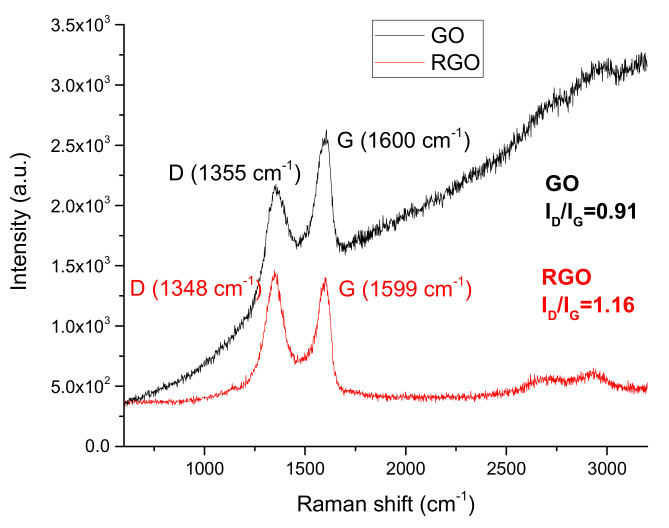
Raman spectra of the GO and RGO samples.

Raman spectroscopy was used to estimate the degree of GO reduction in the RGO sample and to find structural differences between them (Figure 5). The main bands for the graphene derivatives appeared at about 1350 cm^−1^ (D band) and about 1600 cm^−1^ (G band), respectively. The G peak corresponds to the sp^2^ hybridization of the carbon network and is attributed to the first order scattering from the E_2g_ phonon modes in the Brillouin zone. Moreover, it originates from the stretching of the sp^2^ carbon pairs in both the rings and chains [46]. The D peak corresponds to the breathing mode of the aromatic rings (disorder-induced modes existing because of structural defects). Therefore, the intensity of the D peak is used to measure the degree of disorder. The positions of the D and G bands were similar for both the GO samples. The ratio of the intensities of the D and G bands (I_D_/I_G_) determines the reduction degree of GO and provides information about the changes in the sp^2^ and sp^3^ hybridization domains in the carbon lattice [47]. The I_D_/I_G_ values for the GO and RGO were 0.91 and 1.16, respectively. This parameter increases with the increase in the degree of disorder. A prerequisite for the presence of the D peak in the RGO is the presence of sp^2^ domains, which are partially defected (broken structure of the conjugated π–π bonds). The increase in the intensity of this peak can be therefore related to the presence of a greater number of small sp^2^ domains and is largely related to edge defects—the edge effect is higher for smaller flakes created during the reduction. Based on this information it can be stated that GO was slightly reduced after the reaction with ascorbic acid, which led to a partial restoration of the graphite structure.

### 3.2. Composite Characterization

#### 3.2.1. X-Ray Diffraction (XRD)

**Figure 6:**
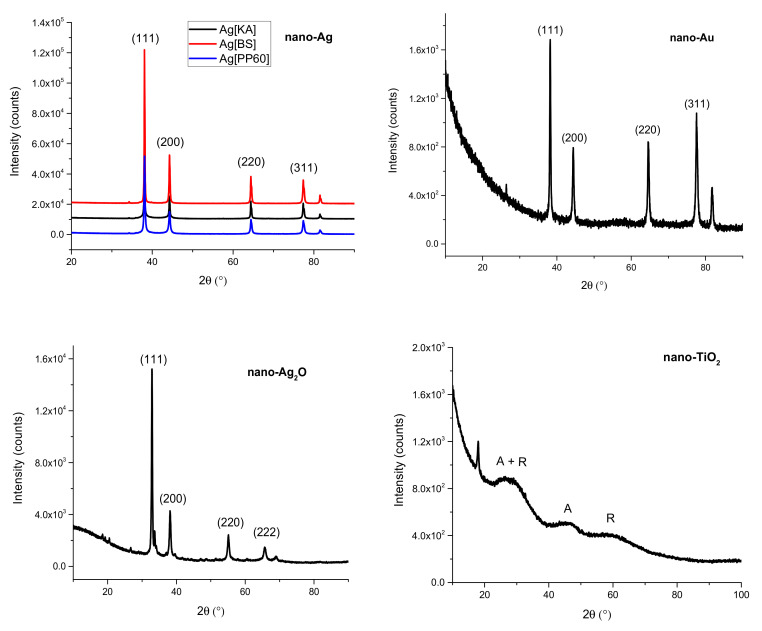
X-ray diffraction (XRD) spectra of nano-Ag—Ag[KA], Ag[BS], and Ag[PP60]—nano-Au, nano-Ag_2_O, and nano-TiO_2_ particles.

Based on XRD measurements (Figure 6), it can be stated that the average size of Ag[KA] was 33 ± 6 nm—this is the size of the coherent regions of Ag, not the grains themselves. A pure, single-phase Ag was obtained. The determined network constant was 4.089 Å. The XRD measurements for Ag[BS] also indicate that pure, single-phase Ag was obtained. The network constant determined here was 4.088 Å, and the average crystallite size was 42 ± 12 nm. The average crystallite size for Ag[PP60] was 20 ± 4 nm—the smallest value obtained. The sample was single-phase with a network constant of 4.008 Å. The sizes of the crystallites in each case were calculated using the Scherrer method. H, k, l indexes marked in Figure 6 were compared with Vanaja et al. (2013) [48], Yang at al. (2019) [49], Reddy et al. (2014) [50] and Sutrisno et al. (2018) [51].

The XRD measurements indicate that nano-Au particles with a determined network constant of 4.079 Å were obtained. The average crystallite size calculated by the Scherrer formula was 17 nm. The network constant calculated from the Scherrer formula for Ag_2_O nanoparticles was 4.714 Å, and the average crystallite size was about 59 nm, with a defective crystal structure. According to the XRD analysis of nano-TiO_2_, an amorphous material was obtained in which traces of crystallization nuclei of anatase (A) and rutile (R) can be observed.

#### 3.2.2. Raman Spectroscopy

**Figure 7:**
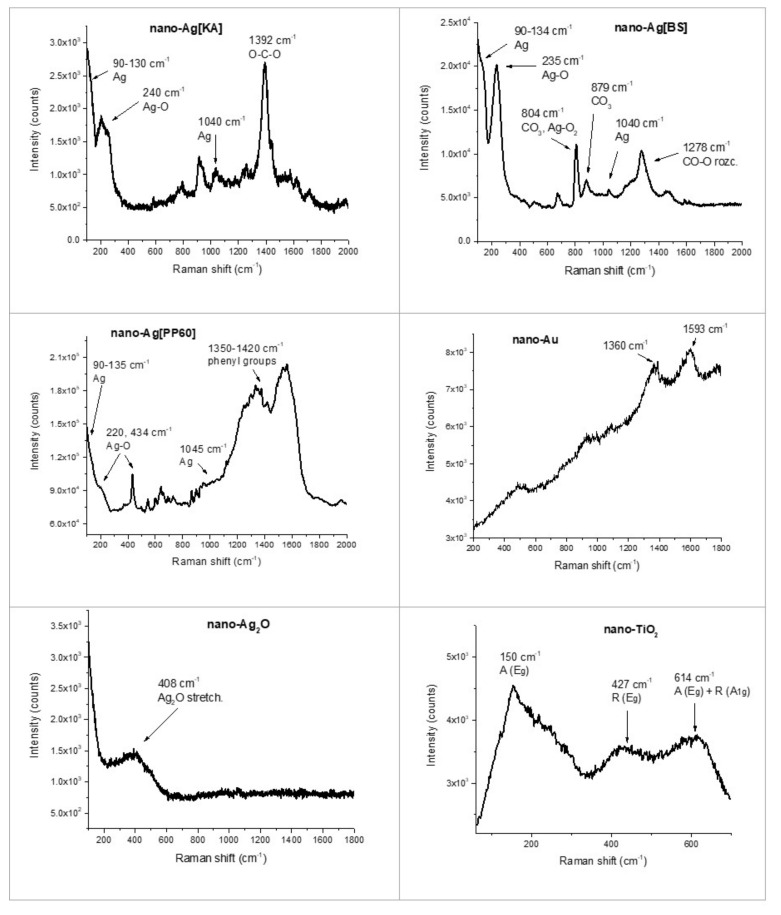
Raman spectra of nano-Ag—Ag[KA], Ag[BS], and Ag[PP60]—nano-Au, nano-Ag_2_O, and nano-TiO_2_ particles.

The Raman spectra (Figure 7) for all the three of the silver samples show characteristic peaks in the range of 90 to 130 cm^−1^ (Ag mode), at approximately 240 cm^−1^ (Ag-O mode), and at approximately 1040 cm^−1^ (Ag mode). There is also a signal for Ag[KA] indicating the presence of carbonates, which is the result of exposing the material to atmospheric conditions [52,53]. The Raman spectrum for Ag[PP60] also indicates that the sample contains polyphenol groups, as this chemical was used as a silver ion reducer. This is demonstrated by the peaks in the range of 1350 to 1440 cm^−1^, corresponding to the stretching vibrations of the COO groups and phenyl rings [54]. Based on the analysis, it can be concluded that the polyphenol present in the sample acts as an inhibitor of agglomerate formation and as a stabilizer of silver nanoparticles; this corresponds with the XRD and SEM measurements (Section 3.2.4). The Raman spectrum of the nano-Au shows peaks, which are characteristic of gold nanoparticles at 1360 and 1593 cm^−1^ [55]. The band in the Raman spectrum for nano-Ag_2_O at above 400 cm^−1^ can be attributed to the stretching vibrations (Ag-O) of the nanoparticles [56]. The Raman spectrum of the nano-TiO_2_ particles indicates the presence of nuclei of the anatase (A) and rutile (R) phases; the respective bands are marked on Figure 7 [57,58].

#### 3.2.3. EDS Spectroscopy

**Figure 8:**
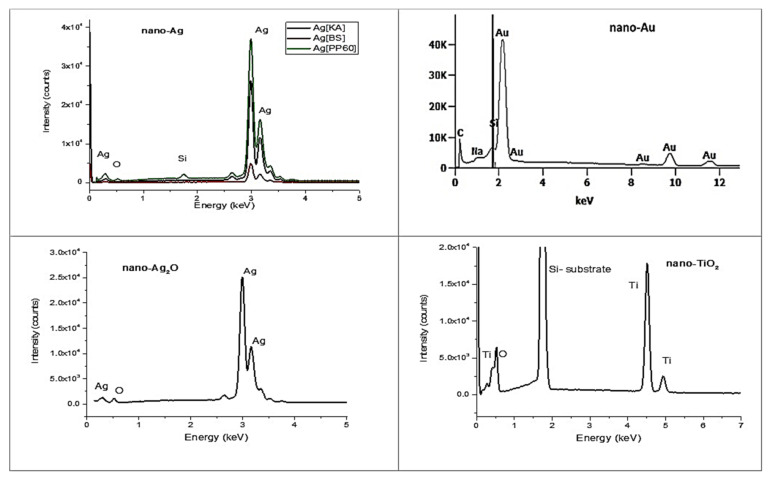
EDS spectra of nano-Ag: Ag[KA], Ag[BS], and Ag[PP60], nano-Au, nano-Ag_2_O, and nano-TiO_2_ particles.

EDS measurements (Figure 8) made of each sample confirmed the chemical purity of the produced nanoparticles. The peaks which are marked in the spectra correspond to the atoms of which each material is composed. In addition to the proper atoms, there are signals from the silicon (Si) that is present in the substrate.

#### 3.2.4. SEM Microscopy

To visualize the morphology of pristine nanoparticles, they were produced without the presence of GO/RGO. However, the synthesis conditions (molar ratios, time, temperature) were kept as for the respective synthesis conducted in the presence of GO/RGO.

**Figure 9:**
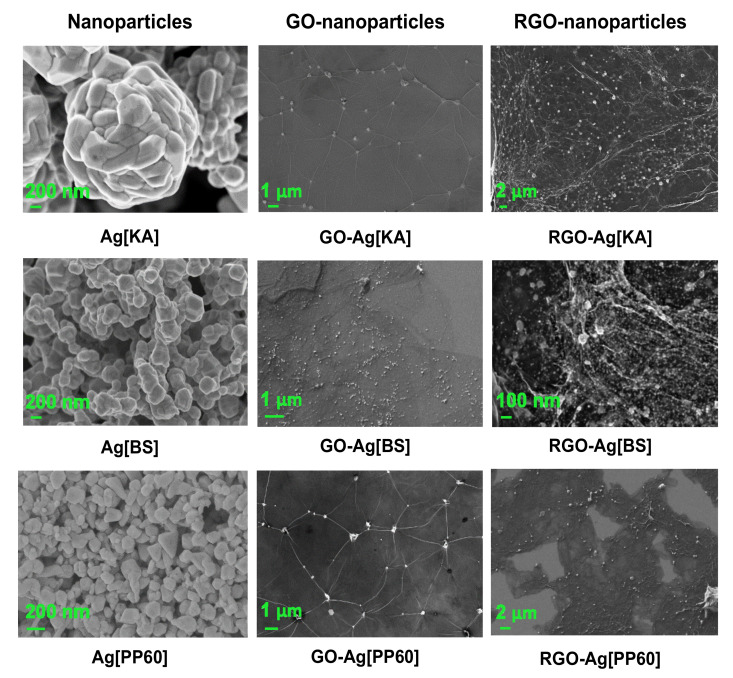
SEM images of nano-Ag—Ag[KA], Ag[BS], and Ag[PP60] nanoparticles—and their composites with GO and RGO. Different magnifications were used for the best presentation of the morphology of each material.

The SEM images in Figure 9 show that ovally shaped Ag[KA] formed agglomerates about 2 μm in diameter. They consisted of smaller, nanometer-sized particles. The sizes of the roundish shaped Ag[BS] were about 200 nm; also, they did not form large agglomerates. The silver particles obtained by the reduction of silver salt with polyphenol (Ag[PP60]) were the smallest and of least uniform shape among those produced: approximately 100 nm. In addition, these nanoparticles did not form agglomerates. Ag[KA] particles deposited on GO flakes formed both small agglomerates and fine nanoparticles. The SEM images show a good distribution of silver nanoparticles on the RGO flakes, despite the appearance of agglomerates in some places. It can also be stated that the GO composite with Ag[BS] nanoparticles and a good distribution of flakes was obtained. The distribution of Ag[BS] was more homogeneous than that of Ag[KA]. The SEM images indicate that an RGO-Ag[BS] composite was obtained in which the silver nanoparticles were distributed evenly throughout the sample volume and did not form agglomerates. The SEM images also show the presence of Ag[PP60] particles on the GO flakes, which formed small agglomerates, comparable to those present in the GO-Ag[KA]. Similarly, in the RGO-Ag[PP60], Ag particles were present in the entire volume, though they formed larger agglomerates than in the case of Ag[BS]. The reason for the formation of agglomerates could be the high affinity of polyphenol molecules for graphene flakes, which meant that the polyphenol could not ensure effective stabilization of Ag nanoparticles alone, as it was observed for Ag[PP60] without the presence of flake graphene. The affinity of polyphenol to graphene flakes is due to the presence of the oxygen functional groups and carbon aromatic rings that occur in these two materials.

**Figure 10:**
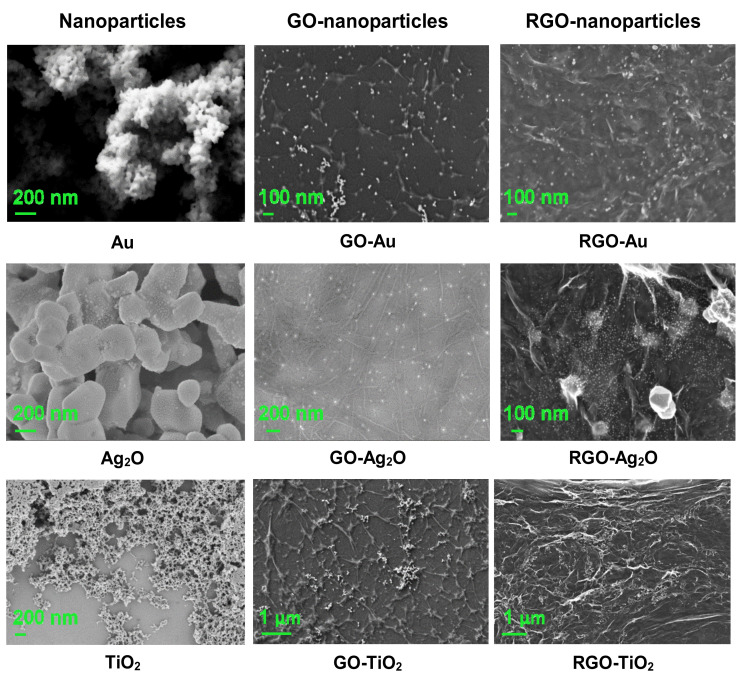
SEM images of nano-Au, nano-Ag_2_O, and nano-TiO_2_ particles and their composites with GO and RGO. Different magnifications were used to best present the morphology of each material.

The SEM images (Figure 10) indicate that gold nanoparticles of small sizes—far below 100 nm—were obtained. The Au nanoparticles were evenly distributed on the GO flakes and did not form agglomerates. Very good, homogeneous distribution can also be seen on the RGO flakes. The particles are nanometer-sized, so it is difficult to determine their diameter based on SEM images.

The morphology of the nano-Ag_2_O was also examined by the SEM technique. The images indicate that the grain size of the material was 200–400 nm, but smaller dots can also be observed. The resulting SEM images of composites show that nano-Ag_2_O particles have good distribution on both the GO and the RGO flakes, creating small nanometer-sized dots and larger agglomerates, especially in the RGO. Based on the SEM images, it can be estimated that the diameter of the TiO_2_ particles is well below 50 nm. The nano-TiO_2_ particles created a very good distribution on the GO and RGO flakes—they did not form agglomerates and were uniformly present in the entire volume of composites.

## 4. Conclusions

In this paper we described in detail the effective methods of preparing composites consisting of graphene flakes (GO and RGO) and inorganic nanoparticles—Ag, Au, Ag_2_O, and TiO_2_—of a known biological activity. Moreover, three different routes of silver nanoparticle synthesis were compared in the creation of the desired homogenous composites with flake graphene. The forms of graphene used (graphene oxide and slightly reduced graphene oxide) are known to be nontoxic towards human cells, for example, mesenchymal stem cells, a finding that was confirmed in our previous work [7]. This manuscript provides a broad morphological and chemical description of each of the inorganic particles and composites produced in this study. Obtained results allow us to state that the described synthesis protocols enable the fabrication of GO and RGO composites, decorated with evenly distributed NPs of different origin. The idea of this work was to introduce effective methods of production of these composites that could be used for future biomedical applications such as antibiotics, tissue regeneration, anticancer therapy, or bioimaging. Our next work, connected to this paper, will concern a biological study of these composites with human mesenchymal stem cells.

## Figures and Tables

**Table 1:** Concentration of atoms in the structure of GO and RGO obtained from XPS survey spectra.

Area	% Atoms	
	GO	RGO
C 1s	65.3	79.5
O 1s	33.0	17.8
N 1s	1.5	1.1
Other	0.2	1.6

**Table 2:** Results of XPS C1s spectra deconvolution of GO and RGO samples.

Chemical Group	Position (eV)		% Atoms		Literature
	GO	RGO	GO	RGO	
C=C sp^2^	284.46	284.61	10.22	43.70	[36,37,38,39]
C–H	284.91	285.44	8.98	7.40	[36,40]
C–C sp^3^	285.38	284.99	5.09	11.30	[36,37,38,39]
C=C–O, C–N	285.84	285.81	2.29	3.40	[40,41]
C–OH	286.34	286.44	12.69	7.80	[36,37,39]
(CC)>O	286.81	287.04	24.95	10.40	[36,37,38,39]
C–O–C	287.26		16.87		
CC=CO	287.82	287.65	5.22	5.00	
O–CC=CO	288.43	289.01	4.11	4.90	
CO_3_^2−^	289.10	285.51	1.66	1.40	[42]
π–π shake-up	−	290.94	−	−	[38,39]
Deformed structure	283.08		0.63	−	[36,43]
Deformed structure	283.57	284.09	2.77	−	
Deformed structure	284.02		4.53	4.70	
